# A New Way of Thinking and Talking About Economy: Clinic Managers’ Perspectives on the Sustainable Implementation of a Decommissioning Programme in Sweden

**DOI:** 10.1177/11786329231189402

**Published:** 2023-07-31

**Authors:** Mio Fredriksson, Inga-Britt Gustafsson, Ulrika Winblad

**Affiliations:** Department of Public Health and Caring Sciences, Uppsala University, Uppsala, Sweden

**Keywords:** Decommissioning, sustainability, implementation, clinic managers, funding, healthcare system

## Abstract

Healthcare systems may run into economic problems that may require ‘active’ decommissioning by policy-makers and managers. The aim of this study was to investigate, from a sustainability perspective, the implementation of an extensive decommissioning programme in one of the Swedish regions. Interviews were performed with 26 clinic managers 3 years after initial implementation. Those were analysed inductively, and then discussed based on a model of potential influences on sustainability. Although the programme was only ‘partly sustained’, the result point to a sustained attention to the health system’s poor economy, visible in a great effort by the clinics to maintain their budgets. The most important influences were intervention fit and modifications made at the clinic level (i. innovation characteristics), clinic and health system leadership (ii. context), champions (iii. capacity) and shared decision-making and relationship building (iv. processes and interactions). When implementing decommissioning, it is particularly important to engage managers responsible for the care of patients and clinic budgets from an early stage and to allow them to design approaches based on the staff’s and managers’ detailed knowledge of the situation at their clinics and of the disease area, that is, to achieve fit at the clinics. In this way, the decommissioning approaches can more likely get the character of quality improvement efforts, which increases sustainability and may lead to positive quality outcomes. Despite being unpopular, the study suggests that decommissioning can have positive effects as well, such as creating opportunities to make difficult but necessary changes and fostering increased collegial support during the centralisation of services.

## Background

Fiscal sustainability of health systems is at the top of many political agendas since spending on health is continuously increasing and has outpaced economic growth in most OECD countries.^1^ Crises such as the COVID-19 pandemic adds additional pressure on health systems, which may require rapid responses from policy-makers and managers. Decommissioning – which overlaps with concepts such as disinvestment, retrenchment, rationing and cut-backs is driven by affordability, quality and cost-effectiveness.^2,3^ In contrast to more passive ways of reducing services, ‘active’ decommissioning^4^ refers to deliberate and intentional decisions and actions with the intent to ‘bring about the removal, replacement or reduction of health services’.^3^ It may include activities such as closures of whole organisations or subunits thereof, partial replacements of interventions or reinvestment in cheaper alternatives.^5,6^ Decommissioning is, however, difficult to implement due to perceived as well as real losses that often lead to protests; and failure rates are likely to be higher than in other forms of service change.^3^ Notwithstanding, in an exploratory study by Robert et al,^2^ a number of recommendations for a successful implementation of decommissioning decisions were formulated – establishing a strong leadership team; engaging clinical leaders from an early stage; and establishing a clear rationale for change.

However, Robert et al focus on *initial adoption* rather than *sustainable implementation*. How decommissioning plays out in the long term has been investigated to a limited extent, and Williams et al^3^ conclude that it is difficult to predict the outcomes of decommissioning programmes. In implementation research in general, sustained change is more rarely investigated than initial adoption and implementation.^7,8^ Sustainability may refer to a number of different outcomes.^9,10^ When talking about the sustainability of public health programmes, Scheirer and Dearing^11^ mention continued benefits for patients/consumers, continued programme activities or components, maintained partnerships or coalitions, maintained new organisational practices and procedures, sustained attention to an issue or problem and programme diffusion and replication in other sites. Sustainability of the ideas, beliefs, principles or values that underlie the intervention has also been mentioned in the literature.^12^

Even interventions or innovations that are initially successful often fail to become part of the habits and routines of organisations, and partial sustainability appears to be common.^8,13-17^ In order to further understand sustainable implementation, a number of frameworks have been developed, largely including similar factors considered significant.^9,10,18^ One of the frameworks, presented by Wiltsey Stirman et al,^16^ summarises into 4 broad categories factors identified to be potential influences on the sustained use of programmes and innovations: (i) innovation characteristics, (ii) context, (iii) capacity and (iv) processes and interactions (Table 1). In the present study, this framework (here abbreviated as influences on sustainability, IOS) guided the discussion of the empirical findings from investigating sustainable implementation of an extensive *decommissioning programme* pursued in the context of an acute – but long-lived – financial crisis in one of the Swedish regions responsible for funding and providing healthcare. Thus, in contrast to most sustainability studies that investigate the implementation of programmes or interventions that require *additional resources*, the present study covered a programme that aimed to *reduce spending*, both instantly and more long term. This task may become increasingly relevant because health systems today face many challenges that increase the pressure on existing resources.^19,20^

**Table 1.** Influences on sustainability.

i. Innovation characteristics	• Fit • Ability to be modified/modifications made • Effectiveness of benefit • Ability to maintain fidelity/integrity	ii. Context	• Climate • Culture • Leadership • Setting characteristics (structure; policies) • System/policy change
iii. Capacity	• Champions (internal or external) • Funding • Workforce (staffing, attributes) • Resources • Community/stakeholder support/involvement	iv. Processes and interactions	• Engagement/relationship building • Shared decision making among stakeholders • Adaptation/alignment • Integration of rules/policies • Evaluation and feedback • Training and education • Collaboration/partnership • Navigating competing demands • Ongoing support • Planning

Reworked presentation of the influences presented by Wiltsey Stirman et al.^16^

Harris et al^21^ argue that there is no theoretical guidance or practical advice for an organisation-wide systematic approach to disinvestment in healthcare services, and Williams et al^3^ conclude that the evidence base on which to guide decommissioning policy and practice is weak, and that future work should explore the relationships between contexts, mechanisms and outcomes, and the long-term impact. Thus, the aim of this study was to investigate, from a sustainability perspective, the implementation of an extensive decommissioning programme in one of the Swedish regions. By that, the study contributes to the decommissioning literature by adding a sustainability perspective and to the literature on sustainable implementation by investigating a programme that aimed to reduce spending through ‘active’ decommissioning.

### The decommissioning programme and its context

The responsibility for funding and provision of healthcare services in Sweden lies primarily with the self-governing 21 regions, which raise proportional income taxes on their population (funding approximately 75% of the services; the rest comes from state subsidies and out-of-pocket payments). The regions function as local healthcare systems and have a political leadership that is democratically elected every 4 years (headed by region commissioners and an executive board) and a non-political leadership of public servants such as a financial officer, HR officer and chief medical adviser (headed by the region director). Together, these individuals constitute the region leadership.

Region Dalarna, with its 5 hospitals and approximately 30 health centres, has approximately 285 000 inhabitants. In 2014, the region faced a major financial crisis due to budget deficits for 19 of the past 20 years, and the executive board instructed the region director to develop a detailed plan for how to save, in total, SEK 700 m until 2019 (approximately €74 m at the time). In June 2015, the first ‘readjustment plan’ was accepted in the regional assembly, and in November 2015, the second one (these 2 readjustment plans are referred to as the decommissioning programme). Together, they contained over 100 decommissioning activities: for example, closure of an ambulance station; relocation of satellite primary care centres and specialist services from rural to more urban areas; and a reduced number of hospital beds. They also contained more comprehensive activities such as changing the management of the system and how to work with priorities and planning (see Supplemental Material for details).

## Methods

### Data and measures

The implementation of the decommissioning programme was investigated through interviews with 26 clinic managers (CMs) in Region Dalarna (approximately half of all CMs distributed over 4 divisions). A CM has the overall responsibility for a clinic and is usually responsible for the budget, personnel, quality and safety. The CM generally has a clinical background, such as physician or registered nurse. CMs may be described as ‘hybrid managers’, as they have a professional background but have taken on a managerial role that requires moving between different organisational groups.^22^

The appropriate timeframe to study sustainability is not self-evident, but it should be sufficiently beyond initial implementation, often 2 or more years after implementation.^16^ In this study, we investigated it approximately *3 years after* the first part of the decommissioning programme was implemented; the interviews were conducted between May and August 2018 and lasted between 45 minutes and 1 hour. All participants were asked to participate by e-mail and given written information about the study’s purpose. At the time of the interview, they were also given oral information about the study and gave written informed consent to participate. The study was approved by the regional ethics board in Uppsala (Dnr 2016/504).

A semi-structured interview guide developed by all authors was used; it contained questions about the CMs’ views of the decommissioning programme at large as well as questions focussing on their specific clinics. The questions were formulated based on the literature on priorities and decommissioning and knowledge of the decommissioning process in Region Dalarna. The questions analysed for this study focused on the CMs’ views on the how the decommissioning activities were implemented at their clinics, initially and at present, and their own role as well as the role of the region leadership. It also included questions about insights along the way.

### Analysis strategy

The interviews were first read in their entirety, and passages related to implementation and sustainability were highlighted. These passages were grouped together and analysed inductively, which allowed for a chronological presentation of events; beginning with programme development, continuing with communication of the programme and ending with alignment with the programme’s intent. To ensure credibility and dependability, all authors met for an initiated and detailed discussion on the content of 3 interviews. Discussions continued until consensus was reached about the CMs’ statements.

To further the analysis, the discussion of the inductive findings was guided by the framework by Wiltsey Stirman et al^16^ (the IOS) that summarises factors identified to be potential influencers on the sustained use of programmes and innovations into 4 broad categories: (i) innovation characteristics, (ii) context, (iii) capacity and (iv) processes and interactions (see Table 1). As noted by Scheirer,^12^ among others, sustainability factors cannot be studied in isolation because they interact in complex ways and also interact in different ways over time. Therefore, we do not report or discuss the empirical findings category by category according to the IOS framework, but in a more integrated way.

## Results

### Programme development: Involving the CMs and communicating the changes

Shared decision making among stakeholders and engagement/relationship building were factors repeatedly mentioned by the CMs, factors belonging to the category of *processes and interactions* in the IOS framework. Most of the CMs described that they were highly involved in formulating the decommissioning programme. They were all invited to a formative meeting – where the main actions were determined – and were then prompted to go back to their clinics and work out the details. The CMs saw themselves as the ones who truly understand the services, and what was possible to change or remove. Most of the CMs put a lot of effort into communicating the decommissioning activities to the staff (sometimes individually even though there could be over 100 staff members) and getting the staff ‘on board’. Some of the CMs said this task was tough and mentioned that they had to be clear about the necessity of the changes, which sometimes involved ‘*putting the foot down*’ (#24) and explain that the changes were not optional and why they were necessary. Some of the CMs, however, expressed that this action was something of a balancing act. ‘*If you do it too fast and too hard, then the staff says “I quit”*’ (#22). Some CMs also mentioned that it was their task to inspire and enthuse, thus acting as champions (a *capacity* factor in the IOS framework) and they arranged lectures and study visits to give the staff input. They also saw it as their task to harbour the staff’s fear, anger and frustration.

### A modifiable programme: From closings to updated routines and task shifting

The CMs’ descriptions of the events illustrate that the programme contained many different types of activities and that those were in many cases highly modifiable, a factor sorting under *innovation characteristics* in the IOS framework. One of the most difficult tasks was to close units, which in most cases resulted in negative staff reactions, including irritation, disappointment and resignations. The lack of guidance when closing units was mentioned by the CMs, for example regarding how to dismiss or replace staff, terminate locality leases, electricity and telephone contracts and empty the facilities of furniture and equipment. Some CMs also permanently closed clinics that were already temporarily closed due to staff shortages, which in some instances was described as a relief as they actually had an opportunity to close down units that had constant troubles with staffing, facilities or care quality. A few CMs also merged units, which posed some problems, for example, the merging of different cultures and ways of working. However, by some CMs, the merges were seen as a way of increasing quality, for instance, by creating an environment with a higher level of collegial support (not working alone at many geographically scattered units).

Many decommissioning activities were less drastic. Several CMs described how they reduced their spending by scrutinising all regular expenditures, such as lab tests, X-rays, laundry and meals, and many mentioned that they scrutinised how they handled pharmaceuticals. Other CMs, however, had a hard time finding things to save on. One CM explained that (s)he had no expensive materials to save on, and that the only option was to save on staff.

Furthermore, a CM conveyed that the clinic tried to adhere even more closely to the indications for surgery, that is, to operate on the ‘right’ patients. Some of the CMs also mentioned more permanent changes like new ways of working. A CM mentioned that their clinic now prescribed medications that required less frequent monitoring at a health centre because changes were made to primary care access in their geographical area, as part of the programme. There were also several examples of altered patient pathways, for example, a new patient flow model for diabetes patients, and giving patients return visits the next day with a junior physician instead of admitting them to hospital. Models to reduce length of stay and the number of revisits were also mentioned. One CM exemplified how they reduced the number of visits per patient by organising the visits differently. Now they handed out medical devices to children with asthma at the first visit and taught the children and parents how to use it immediately instead of booking a new visit. Several CMs also spoke about task shifting, which was regarded as a more effective way of using the existing resources and competencies. A quote illustrates this: ‘*If there is something a registered nurse can do instead of a doctor, we do it. If there is something an assistant nurse or secretary can do instead of a registered nurse, we try to do it*’ (#3).

### Intensity: From huge momentum to status quo

The intensity of the programme over time was linked to *context factors* in the IOS framework, such as climate, leadership and setting characteristics. Most of the CMs agreed that the decommissioning process was intense at first, but they said that that the intensity faded over time. One of them explained: ‘*I think it is completely different now. Then it was drastic: “what will happen, will we end up on an even keel?”*’ (#5). The CMs mentioned that they did not perceive the region leadership being as active anymore (they appeared less often at division meetings, et cetera). One of the CMs thought the region leadership had lost speed and was lacking in the strategic planning to reach the goals. Similarly, the clinics returned to having less frequent meetings. At the beginning, many clinics changed the management structure, in particular the frequency of leadership meetings. One CM said that they had frequent meetings with the clinic management team (to test new routines, evaluate, modify and evaluate again), and continued with it until 2017, when they returned to monthly meetings.

### Changes in the local environment: Affecting the ability to maintain fidelity

Many CMs mentioned that it was rather difficult to separate changes linked to the decommissioning programme from other types of processes that were occurring in the local health system over the years of implementation, thus affecting their ability to maintain fidelity and pointing to the need to modify the programme (an *innovation characteristics* factor). One of the CMs explained: ‘*What is difficult is that, the situation back then, the greatest problems in 2015, are not the greatest problems now*’ (#1). One important change on which most of the CMs reflected was the rapidly growing problem with staff shortages (a *capacity* factor the IOS framework) which forced them to reduce bed spaces. They described the difficulty of trying to save money while spending a lot of effort on recruitment. One of the CMs explained how (s)he saw it: ‘*It is a lot of talk about the improved financial situation, but I’ll be damned, that’s because we have so many vacant positions!*’ (#20).

### Organisational support: From fragmented and poor to great

The context factor of leadership was linked to both positive and negative influences on sustainability. While the CMs saw it as their responsibility to implement the decommissioning programme, many of them simultaneously mentioned that their mandate as managers was curtailed when control of spending tightened and the authority to decide over staff numbers and employment was transferred to the central HR function (which was one crucial administrative aspect of the decommissioning programme). However, although some questioned the practice of central HR deciding on all employments, some CMs said this change entailed that they now questioned whether they really needed new staff members or to replace staff that retired. One manager mentioned specifically that (s)he thought they had some overcapacity earlier and that they were now able to adjust.

When the decommissioning programme started, the region had just completed a reorganisation of the local health system, namely by dividing all medical areas into 4 divisions headed by division managers, at the time 4 experienced physicians. Most of the CMs felt they were supported by their division manager when performing the decommissioning activities. One of the CMs expressed: ‘*It has been an enormous support to be in a division, I have to say. It has not been like this previously, when we have made changes. Then you were very lonely as a CM. . .*’ (#21). The CMs mentioned that they tried to support each other in the divisions and that their networks were an opportunity to find out whether someone had previously done something that they themselves planned, and to ask how they approached it. They also discussed such things as salary levels, so people seeking employment could not play clinics against each other. In contrast, they perceived varying degrees of support from the region leadership. Some of the managers said they lacked HR support while others found it adequate (HR was centralised from the clinics to the region during the same period of time as part of the decommissioning programme).

Too little systematic follow-up and feedback was another leadership factor that was mentioned. Many CMs described that after approximately 3 years there were still too little systematic discussion about the region’s priorities. Although it was not the opinion of all CMs, many of them thought there had been too little focus on follow-ups and not enough feedback from the region leadership to the clinics, in particular after the first year. Several of them mentioned that these types of activities must be highlighted and evaluated regularly, otherwise the day-to-day activities at the clinics take over. Furthermore, a common opinion was that they had been given information about the financial effects but not about other types of effects, such as clinical outcomes or patient satisfaction. One manager expressed his/her hesitation: ‘*They say that the quality has not deteriorated, but it is hard to say if it has actually*’ (#23).

### An altered way of thinking about economy?

Associated with the category of *processes and interactions* in the IOS framework, there was a clear adaptation/alignment with the programme’s intentions, but a lack of capacity was also revealed. Many of the CMs mentioned that they were still very cautious about purchases. One of the CMs said: ‘*I believe we have the economic thinking with us still. It lingers. /. . ./ I am always asking if we can afford it. I ask my economist a few times extra, discuss with the clinic management team. Maybe with the division manager*’ (#25). Several of the CMs mentioned that the decommissioning programme led to a new way of thinking and talking about economy, a higher level of awareness about the economic situation and a sharper pressure to keep the budget. Many of them conveyed that they had started collaborating more closely with their controllers. At the same time, the programme revealed a lack of knowledge of economy and the economic systems among some of the CMs. For example, one of them said: ‘*Economy at this level is complex I think. Still, I do not understand half of it to be honest*’ (#8). However, one difficulty the CMs talked about was that they felt some CMs no longer took the task of implementing the decommissioning programme seriously enough, that they seemed to have forgotten about the spending restrictions.

## Discussion

In this section, based on the CMs descriptions we discuss potential influences on the sustainability of the decommissioning programme guided by the IOS framework by Wiltsey Stirman et al,^16^ Table 1. The factors are italicised in the following text. We also discuss the sustainability of the programme in relation to different sustainability outcomes mentioned in the background section.^11,12^

### The sustainability of the programme

It can be concluded that the initial implementation was much more extensive than could be expected, illustrated by the fact that 95% of all decommissioning activities were implemented after approximately 2.5 years.^23^ However, to answer whether the implementation was sustainable is not straightforward. As described in the background section, sustainability may refer to a number of outcomes.^11^ Similar to many other studies, the implementation may be described as ‘partly sustained’. The CMs themselves were unsure about the *continued benefits for the patients* in terms of care quality, although an evaluation found no signs of deteriorated quality.^23^ However, some CMs described that they thought the programme had improved ways of working. Thus, it is worth noting that the effects of decommissioning need not only be negative. There may be opportunities to make difficult but necessary changes, for instance, to close ‘problematic’ service units, improve quality by centralising services, adhere more strictly to rules and reduce overcapacity. Furthermore, the CMs talked about *continued programme activities and components*, such as a reduced number of bed spaces and care facilities. Additionally, some CMs maintained *new organisational practices and procedures*, including new patient pathways, updated admission rules and altered pharmaceutical prescription patterns. However, potentially most important for long-term sustainability of the programme was the *sustained attention to* the region’s *problems* with the economy among many of the CMs and staff members, reflected in the more careful attitude towards spending. Although the decommissioning programme contained over 100 activities, the programme was largely about transforming the culture in the health system, that is, to change the *ideas, beliefs, principles and values* regarding the relationship between the budget and care production and supply,^12^ which appeared to be under way according to the CMs. This culture change may be more important long term than the actual sum of savings, which was estimated to about half of the financial savings-target (approximately SEK 308 m).^23^

### Influences on sustainability

In terms of potential influences on the sustainability of the decommissioning programme, the interviews with the CMs highlighted a close interaction between the programme design and actions taken, that is, between the categories of *innovation characteristics* and *processes and interactions* in the IOS framework. In healthcare, staff and managers are important local stakeholders,^11,12^ and the CMs testified to a high level of involvement in chiselling out what the decommissioning programme would contain, which implies there was *shared decision-making* between the CMs and the regional leadership (what can also be described as an inclusive *project negotiation process*).^24^ The programme details were largely worked out at the clinics, and thus the programme was *modifiable* (for instance, the exact timing of closures and merges, methods for finding savings, saving objects et cetera). This finding suggests there was a process of *adaptation/alignment* with the clinics’ visions and ways of working with the purpose to achieve *fit* within the different contexts in the local healthcare system. It is likely that fit was essential in this case because the programme was itself undesirable and negatively perceived by the staff and patients. Thus, in this case fit can be interpreted as adaptations that enabled implementing the intervention at all, for example, altered patient pathways, task-shifting and modified treatment options. Notwithstanding, there were staff members who did not accept the changes following the programme, did not see the *effectiveness of benefit*, and the participatory approach could not prevent some conflicts from playing out and some staff from leaving.

The interviews with the CMs also indicated a close interaction between the practice setting and its properties, that is, between the *context* and *capacity* in the IOS framework. There were examples of altered *setting characteristics*. For example, the CMs highlighted the establishment of the 4 divisions headed by experienced physicians as crucial. Many clinic management teams also adapted their management structures and increased their meeting frequency. The frequency of meetings within the divisions, as well as between the divisions and the region leadership, was also intensified (cp. *organisational capacity*),^24^ although intensity faded over the 3 years. The divisions, with their internal meetings, enhanced the *engagement/relationship building* among CMs and constituted forums where the CMs could exchange experiences, develop strategy and support each other. At least during the initial phase, these forums resembled so-called *learning networks*.^15,25^ There was, however, knowledge related to decommissioning that the CMs could not find in these networks, for example, on how to practically close down a service. Thus, the CMs to some extent lacked the knowledge or skills necessary to implement the programme activities. Another important example of a lack of knowledge or skills concerned economy, where several CMs mentioned they were not trained to handle large budgets. This finding suggests there was a lack of sufficient human *resources* or human capital.

The region *leadership* was strong in the beginning but weakened over the 3 years, in particular the leaderships’ visibility^15^ to the CMs that lacked continuous confirmation that the programme was a priority. An important part of the weakened region leadership was too little emphasis on process and outcome *evaluations* to illustrate the results.^12^ The CMs were particularly uncertain about whether care quality had been affected. Furthermore, regarding *ongoing support* there were highly fragmented experiences among the CMs, in particular regarding the central HR and economy functions. However, the leadership and support offered by the division managers, that is, the clinical leadership support, was considered sufficient during the entire period. Largely, the CMs themselves functioned as *champions*,^15,26^ that is, individuals with managerial positions that have ‘a sense for the compromises necessary to build support for the programme’ and ‘negotiation skills’.^24^ The CMs conveyed that they tried to influence the thinking about the decommissioning programme in a positive way and understood the need to make compromises to build support for the programme, thus trying to *navigate the competing demands* between the staff and the region leadership.

## The Most Important Influences: Early Involvement and Adaptive Capacity

From a complex systems perspective on sustainability, it follows that the success of some programmes may be less dependent on fidelity to a set of procedures than on the adaptive capacity of the organisation that implements the programme.^16^ It is plausible that the partial success of the decommissioning programme (which can be interpreted as a good result because decommissioning is unpopular and difficult to implement) was largely due to the clinics’ adaptive capacity. The programme and the local context mutually evolved primarily through the clinics’ concretisation of how to save money, and the CMs as ‘hybrid managers’ were crucial to perform the requests by the region leadership while simultaneously considering the professional needs of the staff. Nonetheless, the CMs described a marked drop in the programme’s intensity, in particular in the region leadership’s visibility and strategy work. Thus, in line with best practice as described by Robert et al,^2^ there was initially a strong leadership team that, however, became weaker over the years, indicating that the programme was not continuously refined and improved by the leadership team.^22^

Based on our results, it seems particularly important when implementing decommissioning (which is generally perceived as negative), to engage managers responsible for the care of patients and clinic budgets from an early stage and to allow them to design approaches based on the staff’s and managers’ detailed knowledge of the situation at their clinics and of the disease area, that is, to *achieve fit* at the clinics. In this way, the decommissioning approaches can more likely get the character of quality improvement efforts, which increases sustainability and may lead to positive quality outcomes. However, it is important to point out that decommissioning may have a negative impact on the health services, and it is essential to evaluate how decommissioning activities affect healthcare quality, equity in access etcetera. Furthermore, our findings emphasise that it is not enough only to establish a strong leadership team, but the team needs to continue its leadership far beyond the initial phase of implementation, not least because it has a strong symbolic value, signalling continued importance.

This study has some limitations. Approximately 3 years had passed since the programme started when the interviews took place, and thus there is a risk of recall bias due to, for instance, subsequent events and experiences that may change interpretations or make memories vaguer. Furthermore, because the CMs interviewed were responsible for implementing the decommissioning programme, they may have had an incentive to describe their participation in a favourable way (a form of social desirability bias). To validate the results, the experiences of first line managers and staff must also be investigated. Furthermore, related to this factor is the confirmation bias, when information may be recalled selectively to confirm, for instance, an interpretation of an event or one’s own beliefs. One such potential driver in this case may be that, at the time of the interview, the clinic managers were aware that the region’s economy had improved considerably. This fact may have influenced them to think of the decommissioning programme as successful in terms of balancing the economy.^27,28^ Hence, longitudinal studies may be appropriate to further study the sustainability of this type of programmes and we suggest this approach for future studies. Lastly, because we exclusively interviewed CMs, who may naturally focus on events closely related to the clinic, this could have led to an underestimation of factors of importance situated further away, for example, at the political level or the role of interest groups.

## Conclusions

Drawing on a categorisation of potential influences on the sustainability of new programmes and innovations by Wiltsey Stirman et al,^16^ it can be concluded that the implementation of an extensive decommissioning programme in one of the Swedish regions was partly sustainable after 3 years. There were several examples of continued programme components and activities and new organisational practices and procedures, and perhaps most importantly, a sustained attention to the health system’s poor economy, visible in a great effort by the clinics to maintain their budgets. The most important influences were intervention fit and modifications (i. innovation characteristics), clinic and health system leadership (ii. context), champions (iii. capacity) and shared decision-making and relationship building (iv. processes and interactions).

When implementing decommissioning, it seems particularly important to involve managers who are responsible for the care of patients and clinic budgets from an early stage. They should be given the opportunity to design approaches based on their comprehensive understanding and detailed knowledge of the situation at their clinics and of the disease area, which can lead to implementation fit at the clinics. In this way, decommissioning is more likely to assume the character of quality improvement efforts. This in turn, increases sustainability and may lead to positive quality outcomes. Despite being unpopular, the study suggests there may be positive effects of decommissioning as well, for example, opportunities to make difficult but necessary changes and foster collegial support when services are centralised.

## Supplemental Material

sj-docx-1-his-10.1177_11786329231189402 – Supplemental material for A New Way of Thinking and Talking About Economy: Clinic Managers’ Perspectives on the Sustainable Implementation of a Decommissioning Programme in Sweden Click here for additional data file. Supplemental material, sj-docx-1-his-10.1177_11786329231189402 for A New Way of Thinking and Talking About Economy: Clinic Managers’ Perspectives on the Sustainable Implementation of a Decommissioning Programme in Sweden by Mio Fredriksson, Inga-Britt Gustafsson and Ulrika Winblad in Health Services Insights
