# Supplementary material for: A New Way of Thinking and Talking About Economy: Clinic Managers’ Perspectives on the Sustainable Implementation of a Decommissioning Programme in Sweden
Source: Health Serv Insights. 2023 Jul 31;16:11786329231189402. doi: 10.1177/11786329231189402 (PMC10392155; doi:10.1177/11786329231189402)
Supplement: sj-docx-1-his-10.1177_11786329231189402 – Supplemental material for A New Way of Thinking and Talking About Economy: Clinic Managers’ Perspectives on the Sustainable Implementation of a Decommissioning Programme in Sweden [file sj-docx-1-his-10.1177_11786329231189402.docx]

**SUPPLEMENTARY FILE 1**

**Table 1. Decommissioning activities in plan 1 and 2**

| Service area | Decommissioning activity (driving distance) |
| --- | --- |
| Ambulance services | Ambulance station in Rättvik closed, stationed in Leksand (21 km) |
| Children’s health care | Children’s clinic in Ludvika moved to Borlänge hospital (43 km) |
|  | Children’s clinic in Borlänge hospital moved to Falun (20 km) |
|  | Children’s clinic in Avesta moved to Falu hospital (69 km) |
| Habilitation | The special pedagogical unit Hästberg closed |
|  | Habilitation unit moved from Hedemora to Avesta |
| Surgery | Skönvikt overweight unit closed and moved to Falu hospital (43 km) |
|  | Rehabilitation- and training pools at Falu hospital closed |
|  | Rehabilitation- and training pools at Mora hospital closed |
|  | Skin treatment unit in Malung closed |
|  | Surgery unit in Ludvika moved to Borlänge (43 km) |
|  | Otolaryngology Unit (Ear, Nose and Throat – ENT) in Borlänge closed |
| Women’s health care | Gynecology unit in Avesta moved to Falun (69 km) |
|  | Gynecology unit in Ludvika moved to Borlänge (43 km) |
|  | Gynecology unit in Borlänge moved to Falun (20 km) |
| Medicine | Geriatric unit at Borlänge hospital closed |
|  | Unit 70 and the stroke unit at Mora hospital merged |
| Primary care | Satellite health center in Nås closed/moved to Vansbro (26 km) |
|  | Satellite health center in Furudal closed/moved to Rättvik (37 km) |
|  | Satellite health center in Lima closed/moved to Sälen (26 km) |
|  | Satellite health center in Fredriksberg closed/moved to Sunnansjö (39 km) |
|  | Satellite health center in Söderbärke closed/moved to Smedjebacken (15 km) |
|  | Satellite health center in Horndal closed/moved to Avesta (26 km) |
|  | Satellite health center in Stora Skedvi closed/moved to Långshyttan (16 km) |
|  | Grängesberg and Ludvika health centers merged |
|  | Out of hours services at Falu hospital moved to health center Tisken |
|  | Out of hours services at Borlänge hospital moved to health center Tisken in Falun (Monday-Thursdays 17:00-20:00) (20 km) |
|  | Skogsgläntan rehabilitation center in Särna closed |
|  | Training- and rehabilitation pools (primary care) closed |
| Psychiatry | Adult open psychiatry unit in Hedemora closed, coordinated with Avesta and Falun |
|  | Adult open psychiatry unit in Älvdalen closed, moved to Mora (140 km) |
|  | Psychiatric unit in Leksand moved to Rättvik (21 km) |
|  | Psychiatric unit in Vansbro moved to Malung (44 km) |
|  | Adult psychiatry units in Rättvik and Malung reshaped into nurse-led satellite units with responsibility for Leksand and Vansbro |
|  | Round-the-clock-care for patients with addictions coordinated to Falun |
|  | Adult psychiatry councelling moved to psychiatric clinic in Borlänge |
| Recreation services | Recreation services in Tandådalen closed |
